# Patient partners’ perspectives of meaningful engagement in synthesis reviews: A patient‐oriented rapid review

**DOI:** 10.1111/hex.13279

**Published:** 2021-05-28

**Authors:** Catherine Boden, Anne Marie Edmonds, Tom Porter, Brenna Bath, Kate Dunn, Angie Gerrard, Donna Goodridge, Christine Stobart

**Affiliations:** ^1^ University Library University of Saskatchewan Saskatoon Saskatchewan Canada; ^2^ Patient Partner Saskatoon Saskatchewan Canada; ^3^ School of Rehabilitations Science University of Saskatchewan Saskatoon Saskatchewan Canada; ^4^ Saskatchewan Centre for Patient‐Oriented Research Saskatoon Saskatchewan Canada; ^5^ College of Medicine University of Saskatchewan Saskatoon Saskatchewan Canada

**Keywords:** consumer participation, knowledge synthesis, knowledge translation, patient and public involvement, patient engagement, patient‐oriented research, systematic reviews

## Abstract

**Background:**

A growing literature describes promising practices for patient‐oriented research (POR) generally; however, those for systematic reviews are largely derived through the lens of a researcher. This rapid review sought to understand meaningful engagement in synthesis reviews from the patient partner (PP) perspective.

**Design:**

The review team comprised PPs, librarians, SCPOR staff and academic faculty. We searched OVID MEDLINE and EMBASE, ProQuest Nursing and Allied Health, and core POR websites. Documents describing PP reflections on their involvement in synthesis reviews were included. Screening and data extraction were conducted by two independent reviewers. Thematic analysis was employed to identify themes in the data regarding PP perceptions of engagement in synthesis reviews.

**Results:**

The literature search yielded 1386 citations. Eight journal articles and one blog post were included. Seven studies focused on conducting systematic reviews on a particular health or patient‐related topic to which PP involvement was an important part and two studies focused specifically on the experience of including PP in synthesis reviews. PPs engaged in the review process through a variety of mechanisms, levels and stages of the review process. Three major themes emerged from the data: (1) foster partnerships through team development, (2) provide opportunities for outcomes valued by PP and (3) strengthen the research endeavour.

**Conclusion:**

Fostering partnerships through team development is foundational for meaningful engagement in synthesis reviews. It requires sensitively balancing of various needs (eg overburdening with contributions). Meaningful involvement in reviews has both personal and research benefits.

**Patient Involvement:**

Patient partners were equal collaborators in all aspects of the review.

## 1 INTRODUCTION

A new approach to health‐care research in Canada was initiated in 2011 when the Canadian Institutes of Health Research (CIHR) launched the Strategy for Patient Oriented Research (SPOR). The intent was ‘to demonstrably improve health outcomes and enhance patients' health care experience through integration of evidence at all levels in the health care system’.^1^ Patients were to become co‐investigators within health‐care research, and their voices were to be regarded within a research context formerly dominated by academics and clinicians. In this way, Patient‐Oriented Research (POR) democratizes health research and enables stakeholders, especially patients, to shape the health care system and empowers individuals and communities with the opportunity to influence change.^2^

A patient, according to CIHR, is an overarching term that includes individuals with personal experience of a health issue and informal caregivers, including family and friends.^3^ POR is defined as ‘a continuum of research that engages patients as partners, focuses on patient identified priorities and improves patient outcomes. This research, conducted by multidisciplinary teams in partnership with relevant stakeholders, aims to apply the knowledge generated to improve health care systems and practices’.^3^ Patient partner (PP) involvement in research has been promoted as a means to ensure the relevance of research agendas and questions, facilitate the recruitment of study participants, improve the likelihood of funding success and enhance the dissemination of results.^4^, ^5^, ^6^, ^7^, ^8^ Engagement of PPs in research has proposed value to patients, to researchers and to the improvement of the research process, all of which may ultimately impact health care, policies and outcomes.^2^ Impacts to PPs include increased knowledge of the research process; a sense of empowerment and fulfilment; and the increased ability for patients to advocate for themselves.^2^, ^4^, ^9^ Researchers who engage in POR benefit from gaining a richer understanding of a health condition or disease from the patient's lived experience and an expansion of research opportunities and scope.^2^, ^10^

The operationalization of POR requires further attention as engaging patients in health‐care research is a new team dynamic and tokenism can be a pitfall.^11^ Integrated knowledge translation insists that having all stakeholders and knowledge users represented within POR teams ensures a collaborative team from the outset; one in which all members are equal and one in which all stakeholders are vested in the findings.^12^ Resulting reported improvements to the research process include the identification of research priorities that are relevant to patient needs, more appropriate ‘real world’ research questions and designs, increased credibility and applicability of findings, and improved quality of knowledge translation materials.^2^, ^4^, ^10^ Similarly, a recent scoping review^13^ identified five primary PP roles in research: steering committee membership; advisory committee membership; consultation; co‐design of knowledge translation; and participation in research tasks. Specific examples of steering committee member roles included providing input on design of instruments and tools used in the study,^14^ while advisory roles included assisting with systematic review^15^ and conference planning.^16^ PP consulting activities involved sharing the ways in which intervention would affect patients,^17^ and specific research activities which PPs readily participated in encompassed development of research questions, interview guides, research priorities, data analysis and attendance at study briefings.^18^, ^19^, ^20^ The overarching aims of POR approaches are to optimize health outcomes and quality of life in ways that are important to patients, which ultimately relies on the extent to which patients are meaningfully engaged in the research process.^21^ Existing frameworks^22^, ^23^ describe recommendations to foster meaningful patient engagement in research. Pollock and colleagues^24^, ^25^ focused on methods of involving stakeholders in a specific kind of research, namely systematic reviews. Our review extends this work by investigating how research teams can ensure that PP contributions to synthesis reviews (SRs) are meaningful from the PP’s perspective.

### 1.1 The objective of this study is to better understand best practices in engaging patients in synthesis reviews

Our team synthesized published and grey literature on patients perspectives of meaningful engagement in SRs, employing Hamilton et al's definition: ‘…the planned, supported and valued involvement of patients in the research process within an interactive team and positive research environment that facilitates effective contributions by patients or their surrogates to help to produce important outcomes while benefiting the patients or their surrogates’.^23^ We aimed to answer two questions:

1. What are the characteristics of a review team that create an ‘interactive team and positive research environment that is conducive to effective contributions by patients’?
2. How can research teams conduct their reviews to ensure ‘planned, supported and valued involvement of patients’?

## 2 METHODS

### 2.1 Team composition and training

The review team comprised two PPs (AME, TP), two stakeholders representing the Saskatchewan Centre for Patient‐Oriented Research (SCPOR) (CS, KD), two librarian faculty (AG, CB) and two research faculty (BB, DG). PPs were recruited from a Saskatchewan Centre for Patient‐Oriented Research (SCPOR) patient partner pool. Both PPs were very familiar with the health‐care system although they had never participated in a POR rapid review. An introduction to rapid review methods was provided in the first meeting which included a simulation activity based on identifying chocolate chip cookie recipes within cooking literature. A description of the activity is provided in Supporting information.

All team members were fully involved in all aspects of the review from determining the question to dissemination. Training was integrated into team meetings, including pre‐readings and instructional activities (eg mock data extraction examples), and undertaken by the entire team. Additional informal training was provided as requested. Meetings prior to mid‐March 2020 were held in‐person. In mid‐March 2020, all non‐essential workers in the team's location were required to work remotely due to the COVID‐19 pandemic. All subsequent meetings were held online. The transition to virtual meetings occurred as we finished data extraction. In Table 1, we describe in more detail how PP were involved in the conduct of the review, and team reflections on their involvement.

**TABLE 1:** Guidance for reporting involvement of patients and the public (GRIPP2) checklist.^37^

Aim	To collaboratively involve patients as research partners at all stages in the rapid systematic review research project to ensure PP perspective in the review process and outcomes.
Methods	PPs were recruited through a post on the SCPOR website for research opportunities. Training was integrated into team meetings throughout the review, and additional informal training was provided as needed. PPs were included as collaborators on the research team, participating fully in all aspects of the review from research question refinement to knowledge translation which had equal input for the duration of the project.
Study results	Through their active contributions, PPs helped craft the research question and provided input and insights at each stage of the review. PPs encouraged a team culture of deliberately considering multiple viewpoints during team discussions. PPs lead some stages of the review, specifically the knowledge translation plan and the GRIPP2 checklist content.
Discussion and conclusions	PPs contributed important PP perspectives and lay language. PPs asked provocative and necessary questions at each step of the process and often provided an invaluable lens not only to the research process but to the content as well.
Reflections/critical perspective	Patient Partners: • PPs may want to choose a topic of interest as it is challenging to review content without a personal interest. • PPs received positive feedback from other team members that validated PP contribution during discussions. • PPs were able to ask questions and for clarification during and after meetings. • PPs gained valuable experience in health research and would get involved in future rapid reviews. • PP learned about other team members expertise in their areas of work and perspectives and appreciated a ‘Wonderful opportunity to build a multidisciplinary team that can learn something from everyone’.. • PP had opportunities to step outside of their comfort zone by participating in the review, including being accepted to present at an international conference. • PP should be involved in rapid reviews. PP noted some negatives of their involvement: • Project required extended timelines due to COVID‐19. • Additional and on‐going support may be required if a PP has limited or no experience with rapid reviews or research process. Other team members’: A lack of clarity could have impeded participation without PP’s willingness to ask questions and to communicate needs and workload. Timelines were significantly affected by the pandemic. The willingness of PP to continue with this project in very trying circumstances beyond the agreed time was a significant factor in the completion of this project.

Abbreviation: PP, Patient partner.

### 2.2 Search strategy

We searched the published and unpublished literature for documents describing patients’ reflections on meaningful engagement in SRs. Databases were searched using controlled vocabulary and natural language terms for three concepts: ‘meaningful engagement’, ‘patient‐oriented research’ and ‘synthesis reviews’. The search strategy was developed in OVID MEDLINE and then optimized for the other databases. We searched three databases. The platforms and databases (with earliest coverage date to date searched) were as follows: Ovid MEDLINE(R) and In‐Process & Other Non‐Indexed Citations (1946 to 10 December 2019), OVID Embase Classic+Embase (1947 to 10 December 2019) and ProQuest Nursing and Allied Health (1850 to 11 December 2019). The Salzburg Global Seminar held in 1998, marked the inception of patient engagement in health care with the vision of informed shared decision making.^26^ Therefore, the only limit we applied was to limit the search to articles published from 1998 onward.

The database searches were supplemented with a grey literature search of key websites: INVOLVE, PCORI, Healthtalk.org, NIHR Collaboration for Leadership in Applied Health Research and Care: Oxford, and all SPOR Provincial Support Units. Further, the table of contents of *Health Expectations* (January 28, 2020), a key journal for this topic, were searched. Searches of Google and Google Scholar supplemented the database searches. Appendix 1 for search strategy details.

All citations were uploaded to Endnote citation management software (Endnote X8, Clarivate Analytics) and duplicates were removed. The de‐duplicated citations were imported into Covidence systematic review software (https://www.covidence.org/home).

### 2.3 Study selection

We included documents which described or reflected on PP perspectives of meaningful engagement in SRs. PPs had to have participated in the conduct of a synthesis review, but there was no restriction on the level of engagement. The document had to focus on meaningfulness from the patient's perspective. Thus, we included only reflective articles and grey literature (eg reports) written by or in collaboration with patients documenting their experiences on SRs. Documents describing patient engagement in SRs where patients were participants in the research and did not contribute to the planning, conduct or dissemination, were excluded. SRs include systematic reviews, scoping reviews, realist reviews, meta‐syntheses, meta‐analyses, qualitative reviews, health technology assessments and clinical practice guidelines. Patient engagement in other kinds of reviews (eg narrative reviews) or research was excluded. We were interested in methodologically rigorous reviews which are more typically employed to guide research agendas (eg scoping reviews) or practice/policy (eg systematic reviews, realist reviews) specifically.

All members of the team contributed equally to the screening process. Screening was conducted by pairs of two independent reviewers using a screening form developed and piloted a priori. First, the titles and abstracts of citations were reviewed. Then, the full text of citations deemed of possible interest was retrieved and reviewed. Disagreements were resolved through consensus at both stages. Covidence software was employed to facilitate the screening process.

### 2.4 Data extraction

We employed Hamilton et al's definition^23^ of meaningful patient engagement as the framework for extracting data about PP engagement in the review process. Data were extracted by pairs of independent reviewers using an extraction form that was developed and piloted a priori (Supporting information). All members of the team contributed equally to extracting the following data:

- Descriptive data about the publication (ie authors, publication year, journal, country of corresponding author, country in which study was conducted, sponsorship and funding);
- Descriptive data about the study (ie overall aim and purpose of the study, total number of authors, number of PP authors, study design, synthesis review team composition, PP engagement and training);
- Qualitative data regarding the mechanisms for PP engagement and training, and PP perceptions of that engagement. Excerpts from the study pertaining to PPs involvement in a systematic review were coded for voice (patient or research team voice).

### 2.5 Data synthesis

Frequencies were calculated for descriptive data about the publication and the study. Qualitative data were analysed using thematic analysis as described by Braun and Clarke.^27^ Initially, all team members independently coded the data to become fully familiar with the data set. This initial coding ensured deep understanding of the dataset by each team member but yielded eight unique coding schemes. The extracted data by the POR team were then coded and themed using NVivo version 11 by the Canadian Hub for Applied and Social Research (CHASR; formerly the Social Science Research Laboratory) as time constraints introduced by the COVID‐19 pandemic made it more difficult for the team to meet and collaborate. The themes were refined and interpreted through team discussion, leading to a deeper understanding of the nuances of and relationships among the themes and subthemes. Practical recommendations for creating an environment in which PPs perceive they can and have made meaningful contributions to the review were identified.

## 3 RESULTS

### 3.1 Study selection

**FIGURE 1:**
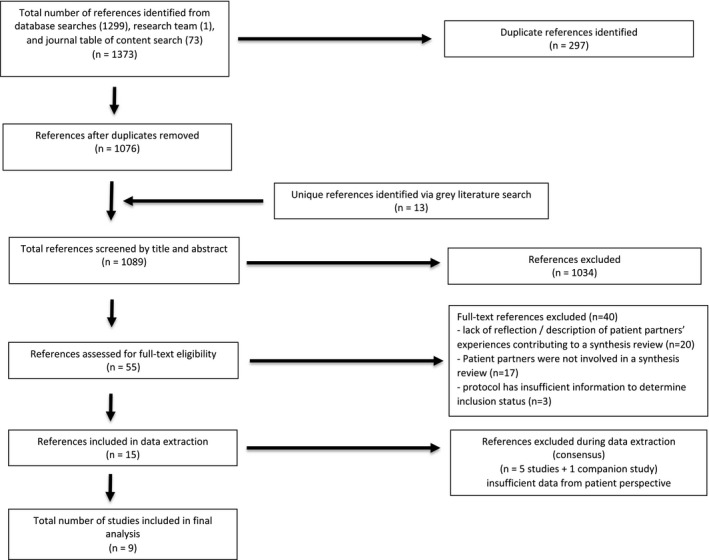
PRISMA flow diagram

The database searches yielded 1386 citations (1373 from databases, 13 from the grey literature search). After removing 297 duplicates, 1089 citations were screened. Forty articles were excluded upon review of the full text for the following reasons: lack of reflection on PP experiences contributing to a synthesis review (n = 20); PPs were not involved in a synthesis review (n = 17); and the protocol had insufficient information to determine inclusion status (n = 3). Five studies and one companion study were removed during data extraction due to insufficient data from patient perspective. Eight journal articles and one blog were included (Figure 1).

#### 3.1.1 Characteristics of included studies

Studies employed the following methods: questionnaires/survey (2), basic interpretive (1), SR (1), Delphi (1), case study (1), blog post reflection (1) and unclear (1). An average of 1.3 (range 0‐6) patient partners were authors on the articles. Corresponding authors were located predominantly in the UK (6 articles) with one each from Germany, Norway and Spain. Eight studies reported funding and one study did not have a funding statement. Four of the nine reports included in this study include PPs as authors.^15^, ^28^, ^29^, ^30^ Only four studies explicitly used the ‘voice’ of the PP with direct quotes from the PP embedded within the article.^15^, ^28^, ^30^, ^31^ One study ^29^ summarized reflections from PPs that were part of the engagement activities, but direct patient quotes were not used. One study used exclusively the researchers’ voices to describe events and experiences of the PPs and their participation in the review.^32^ In the remaining studies, it was less clear as to who was lending their voice to the interpretation of events. One study^33^ did not include any PPs as authors, so the voice portrayed was likely that of the researchers’. Two studies^34^, ^35^ also appear to be presented in the voice of the researchers. However, one ^28^ had PP authors, so it is possible they lent much to the interpretation and summary. Characteristics of the nine included studies are provided in Table 2.

**TABLE 2:** Characteristics of the included studies

Author, year	Journal name / source	Country of corresponding author	Total number of authors	Number of patient partner authors	Sponsorship / funding	Reporting of Ethics Approval/Guidance for PP involvement.	Overall aim and purpose of study	Study design
Bayliss et al, 2016^28^	Research Involvement and Engagement	UK	10	6	FP7 HEALTH (Euro‐TEAM) and by Riksbankens Jubileumsfond (The Swedish Foundation for Humanities and Social Sciences)	Did not report	‘The aim of this paper is to inform the evidence base on effective ways of involving patients in a qualitative meta‐synthesis. This process is evaluated and reflected by patient research partners (PRPs) who provided accounts of their experience’.	open‐ended questionnaire
Brütt et al, 2017^33^	Z. Evid. Fortbild. Qual. Gesundh. wesen (ZEFQ)	Germany	7	0	German Federal Ministry of Education and Research	‘The study was conducted in accordance with the Helsinki Declaration’	‘We focused on how patients could be meaningfully involved in our systematic review to complete the review protocol. The aims of the present study were a) that patients contribute and prioritize clinically relevant outcomes of meta‐cognitive interventions, and b) that they evaluate a patient workshop and give their perceptions of research involvement’.	questionnaire (cross‐sectional survey) & focus group
Coon et al, 2015^29^	Health Expectations	UK	9	2	NIHR HTA programme and NIHR CLAHRC South West Peninsula at the Royal Devon and Exeter NHS Foundation Trust	Did not report	‘Objectives: (i) Describe end‐user involvement in a complex mixed‐methods systematic review of ADHD in schools, (ii) reflect on the impact of end‐user involvement, (iii) highlight challenges and benefits experienced and (iv) provide suggestions to inform future involvement’.	Unsure
Jamal et al, 2015^31^	Health Expectations	UK	6	0	NIHR Public Health Research	Did not report	‘This study describes the process and impact of consulting with a young people's advisory group to inform decision making in a systematic review on the effects of schools and school environment interventions on children and young people's health’.	Case study
Myrhaug and Hansen, 2019^30^	The BMJ Opinion (BMJ blog)	Norway	2	1	No funding statement included.	Did not report	Researcher and patient perspectives’ on how they collaborated on a systematic review	non‐research ‐ reflection in blog post
Serrano‐Aguilar et al, 2009^35^	Social Science and Medicine	Spain	6	0 (with some uncertainty)	Ministry of Health and Consumption of Spain in co‐operation with the Fundacio ´n Canaria de Investigacio ´n y Salud (FUNCIS)	Reported ethics approval for patient participation in rounds of Delphi employed to inform research.	‘The aim of the present study was to involve patients in developing the early stages of a systematic review process’.	Delphi method
Staniszewska et al, 2019^34^	The British Journal of Psychiatry	UK	10	0	NIHR CLAHRC West Midlands and NIHR CLAHRC North Thames at Bart's Health NHS Trust	While the study received formal ethics approval, the researchers noted in their conclusions that ‘clear guidance on the ethical dimensions to PPIE is needed’	‘To conduct a systematic review of published literature, identifying key themes for improving experiences of in‐patient mental healthcare’.	Scoping / systematic review combined
Troya et al, 2019^32^	Health Expectations	UK	6	0	Keele University ACORN studentship; Santander Bank and The Allan and Nesta Ferguson Charitable Trust; West Midlands CLAHRC		‘Critically reflect on the process, potential impact and identify challenges and opportunities in involving robust PPIE [Patient Public Involvement Engagement] in a doctoral study’.	Case study
Vale et al, 2012^15^	Systematic Reviews	UK	6	3	Medical Research Council and UK Department of Health NCC RCD Postdoctoral Research Scientist in Evidence Synthesis Award	Did not report	‘to evaluate [patient partner] involvement with the aim of informing the practice of patient involvement in future systematic reviews’	Basic Interpretive

Abbreviation: CLAHRC, Collaboration for Leadership in Applied Health Research and Care; NIHR, National Institute for Health Research.

Only two of the included studies focused specifically on the experience of including PPs in systematic reviews (including one meta‐synthesis).^28^, ^30^ Seven of nine studies were reviews conducted on a particular health or patient‐related topic to which PP involvement was an important part.^15^, ^29^, ^31^, ^32^, ^33^, ^34^, ^35^ The latter included reflections by the PPs or the research team on engagement in the review process.

Research teams provided formal and informal training in synthesis review methods to PPs, which occurred at specific points in the review or, in one case, interspersed through the project. Four studies mention training specific to synthesis review methods.^15^, ^30^, ^32^, ^33^ Three studies provided some form of training but it was unclear if supports were specific to SR methodology: monthly training sessions,^31^ written instructions^28^ and informal training and support.^29^ Two studies were unclear or did not report on training for PPs.^34^, ^35^

PPs engaged in the review process through a variety of mechanisms (workshops, interviews, rounds of Delphi, email, etc). The level (consultation to full collaboration) and frequency (single stage of the review to continuous engagement throughout the conduct of the review) of PP engagement varied across studies.

### 3.2 Synthesis

Three major interrelated themes emerged from thematic analysis: 1) foster partnerships through team development; 2) provide opportunities for outcomes valued by PP; and 3) strengthen the research endeavour. Below we describe the themes and subthemes. The relationship of the themes and subthemes to our two research questions is provided in Table 3. A foundational element to meaningful patient engagement in SRs is fostering partnerships through team development, which in turn leads to generally positive experiences and benefits to the PP themselves, and ultimately strengthens the research process, outcomes and potential impact.

**TABLE 3:** Relationship of themes and subthemes to the two components of the research question

Theme	How can research teams conduct their reviews to ensure ‘planned, supported and valued involvement of patients’?	What are the characteristics of a review team that create an ‘interactive team and positive research environment that is conducive to effective contributions by patients’?
3.2.1 Foster Partnerships Through Team Development	3.2.1.1 Provide Accessible Training	Sufficient training is provided so PP know they are full team members.
	3.2.1.2 Build Supportive Relationships	A respectful team environment that allows PPs to know they can openly voice opinions and experiences.
		A research culture sensitive to PP potential.
		A team environment that mitigates risks to PP and is attentive to team emotions.
	3.2.1.3 Clarify Roles	A team in which all members, particularly PP, understand their role throughout all stages of the review process.
	3.2.1.4 Balance PP and Research Team Expectations and Opportunities with Resources	A team in which expectations for PP involvement align with the realities of finite resources, and is understood by all members.
3.2.2. Provide Opportunities for Personal Outcomes Valued by PP		3.2.2.1 Provide Satisfaction and Sense of Accomplishment. Engagement in which PP experience a sense of satisfaction and accomplishment.
		3.2.2.2 Examine Beliefs and Expand Learning. Engagement in which PP can examine beliefs and expand their learning.
3.2.3 Strengthen the Research Endeavour		3.2.3.1 Influence Research Priorities. Team environment which enable PP to influence research priorities
		3.2.3.2 Enhance Research Relevance and Quality. Team environment which enable PP to enhance research relevance and quality.

#### 3.2.1 Foster partnerships through team development

Subthemes of this theme describe how research teams can conduct their reviews to enable meaningful engagement by PP and the characteristics of teams that create positive research environments.

##### Provide accessible training

Accessible and respectful training that familiarized the PPs with the research process was a key component of ensuring PPs felt they were full partners in the research process:

‘The presentations and design of the materials took great care to unravel the complex world of research acronyms and concepts and explain complex ideas simply but without dumbing down. That made me feel that we were equal partners in a really important piece of work’. ^15^ [patient partner]

Despite the importance of tailoring the information to PPs requirements, it can be a difficult task to achieve, as this researcher points out from their experience:

‘The patient group was quite heterogeneous, with participants having different disease‐related impairments and diverse experiences with research projects. Adapting the information given to those different needs remains challenging’. ^33^ [research team]

##### Build supportive relationships

A critical element of PP engagement in SRs was building supportive relationships between PPs and researchers. In the absence of adequate time and support, there is a risk that the relationship becomes more consultative than collaborative. Ideally, relationships should allow PPs to feel they can openly voice their opinions and experiences and that their opinions will be respected:

‘What was difficult was being part of a systematic review that did not support my prior belief. But this was greatly helped by the respect afforded to me from the review author group and the time we spent jointly discussing why the findings were inconclusive…’^30^ [patient partner]

‘This ended up being a matter of debate between us, but because of the training I had undergone and our established good working relations, we were able to understand each other’s perspectives and reach agreement.’^30^ [patient partner]

Additionally, from the research team perspective, taking the time to build relationships ensured PPs could reach their full potential as a team member, fulfilling an important role, rather than just a title:

‘…build research cultures sensitive to PPIE’s potential contribution and develop the expertise needed to avoid tokenistic involvement.’^32^ [research team]

A number of factors relating to the development of productive relationships were identified. One review^32^ outlined a variety of supports that were provided to their PPs to actively mitigate negative emotions among PPs. These included logistical support that accommodated time and location needs for PPs; training support with accessible materials; and support for PPs’ physical and emotional well‐being.

In regard to supporting well‐being, it was noted there were instances in which the researchers took steps to mitigate the risks PPs may have experienced. These included the following:

1. Power relations: ‘Participants were therefore protected from the influences of the group and the prestige or power of certain participants, suggesting that their opinions and proposals might be more realistic’.^35^ [research team]
2. Work burden: ‘One example of adjustment in my favour was that my share of reading full text articles was small; I only had to read 14 out of 320 articles and the project leader let me choose which articles to read’.^30^ [patient partner]
3. Negative emotions: ‘The research team took key decisions regarding the role and level of involvement of the PPIE group, including the decision not to include members as co‐researchers/co‐interviewers because of concerns not to cause undue emotional upset’.^32^ [research team]

Being attentive to emotions was discussed by researchers as a challenge when including PPs in systematic reviews as well: ‘…managing the intensity of emotion between individuals with differing viewpoints was challenging at times’.^29^ For example, in one review, a PP highlighted the need to be ‘better informed about the sorts of discussions that would take place…[as] the clinicians are going to be blunt and scientific in their approaches and not the normal ‘bedside manner’ we might be used to as patients!’^15^

Being attentive to PP emotions appeared to be handled differently by different teams. Not all researchers took steps to fully avoid negative emotions among PPs. For example, in Coon et al,^29^ the PP experienced an emotional toll when participating in the systematic review, yet felt her involvement in the review was important:

‘CS reflected that reading the draft chapters had been an emotional experience for her…reading about the difficulties that people with ADHD face in black and white reminded her of the costs of ADHD to her family and was painful, but nonetheless, she was pleased to be involved as the drafts held the potential to help others cope with ADHD’.^29^ [patient partner]

##### Clarify roles

Ensuring PPs have an understanding of their role on the team was deemed to be important.

These roles appeared to be assigned on the whole, although it was unclear whether PPs contributed to defining the nature and extent of their role.

‘Further discussions took place with the group to clarify roles and refine levels of participation in order to avoid overburdening. PPIE members did not offer suggestions with regard to the structure of their involvement’.^32^ [research team]

It appears, however, PPs found the assignment and clarification of roles useful in order to understand the expectations of team members.

‘Overall my experience working on this review was positive. The expectations of me as a co‐author were clear, I had time to complete my work’.^30^ [patient partner]

‘I remember we were sent the explanatory materials and offered a Skype or phone call talk with the researcher/the specialist, which was very helpful in letting me know what the research team expects from me’.^28^ [patient partner]

It was identified that clarifying the PP’s role should be reaffirmed throughout the project, not only at the outset, as on‐going communication could avoid surprising PPs with tasks or new information.

‘One result of this was that CS did not know she was being asked to comment on drafts of reviews for which she was a project team member. She did not connect the application she had been involved with previously with the qualitative draft reviews when asked for comments. She was happy to give comments, but was astonished to learn upon consultation for this study that she had been a named team member’.^29^ [research team]

##### Balance PP and research team expectations & opportunities with resources

There were several areas identified as potential sources of tension, which needed to be balanced. Researchers indicated struggling with finding a balance between providing PPs enough and appropriate opportunities to adequately contribute while avoiding overburdening them.

‘…and whilst we aimed to achieve a balance between people feeling involved and burdened by the involvement, this might have reduced the sense of being involved for some people’.^29^ [research team]

Ensuring adequate time for training and relationship building needed to be balanced with project timelines and other deadlines. Adequate training must be provided to ensure PPs have the knowledge and skills to participate fully, while ensuring it is not delivered condescendingly. Time is also needed to build relationships in which mutual respect can naturally develop. Ensuring adequate time to incorporate important learning may be compromised by tight research deadlines.

‘Obtaining academic and a parent viewpoint on the drafts of the report was seen as invaluable by the researchers, helping to validate and fine‐tune the conclusions and recommendations for future research in particular. It was, however, difficult to allocate time and attention to make the most of end‐user input towards the end of the project when deadlines were tight’.^29^ [research team]

Therefore, decisions made by researchers early in the project can have a profound effect on the roles and engagement of PPs over the course of a project;

‘… decisions made by professional researchers at the outset of a study have a cumulative and significant influence on the potential for [patient partner involvement] to impact on a study and that involvement is more difficult to achieve once studies are underway’.^28^ [research team]

Finally, the right balance must be found to ensure both parties benefit from the research and the relationship:

‘Discussing elements of the end‐user involvement with both [PP] highlighted the need to balance the relationship so that all parties consider it to be beneficial’.^29^ [research team]

#### 3.2.2 Provide opportunities for personal outcomes valued by PP

Satisfaction and a sense of accomplishment, as well as validating beliefs and expanding learning, were benefits identified by PP as aspects of meaningful engagement.

##### Provide satisfaction and sense of accomplishment

PPs expressed satisfaction in contributing to research that may help others with similar health conditions.

‘I was very happy for this result, because I believe it might bring hope for cancer survivors struggling with fatigue’.^30^ [patient partner]

‘...I was happy to undertake this work for the topic inspired me and I firmly believed that our findings would be useful in advocating for cancer rehabilitation’.^30^ [patient partner]

PPs also found a sense of accomplishment in participating in a process that could be helpful to other patients.

‘It’s a good feeling from a patient perspective to have contributed to a piece of work which recognizes the after effects of treatment and survivorship issues’.^15^ [patient partner]

##### Examine beliefs and expand learning

Other benefits that PPs highlighted included examining their own beliefs and expanding their learning:

‘I had personal experience and knowledge of multiple patient perspectives and hold a strong positive belief in cancer rehabilitation. I fully expected that the review would find results which favoured multidisciplinary psychosocial intervention’.^30^ [patient partner]

‘I wanted to understand how a systematic review was carried out for I thought this method would be useful in the enormous field of studies being done on blood cancer drugs and treatment’.^30^ [patient partner]

There were a few cases in which PPs needed to step back from their role in the project, for a number of reasons, including losing interest over time, seeing little benefit in participating, and the revelation that evidence was not conclusive:

‘One of the key issues this raised was that as time passed, understandably, one of the Patient Research Partners said that her ‘personal interest in cancer has faded somewhat’.^15^ [patient partner]

‘Reflection from one of the contributors (WP), however, highlighted that although this was an interesting meeting and they were pleased to help, because we were unable to give them clear guidance on which interventions they should be using (or not using), they felt that the meeting had limited benefit for them’. [patient partner]

#### 3.2.3 Strengthen the Research Endeavour

PP and other researchers recognized that engaging PP meaningfully in a systematic review influenced research priorities and enhanced research relevance and quality.

##### Influence research priorities

PPs provided an added perspective to the research process by addressing different priorities for researchers. Once PPs became engaged in the synthesis review, their exposure to the literature provided them the opportunity to see the important gaps in health research, which they were able to identify.

Influencing future research: ‘…the outcome passed my expectations, as I witnessed how the team doing the evaluation realized what information was missing and how future trials could give more detailed and long term information’.^15^ [patient partner]

Reduce redundant or harmful research: ‘I am also aware that few of my fellow patient advocates in the European hematology field are aware of what systematic reviews are and how by identifying best evidence they can reduce harmful and redundant research’.^30^ [patient partner]

Noteworthy is that if these views did not come from PPs directly, the sentiment was expressed by the researcher.

‘The group also identified gaps in the literature from the [systematic review] which they considered as important for patients and public and which require further research’. ^32^ [research team]

‘…partner insights have generated recommendations which may help researchers to involve patients more effectively in future systematic reviews’.^28^ [research team]

##### Enhance research relevance and quality

PPs contributed to the research agenda, outcomes, data analysis, interpretation and dissemination of findings and thereby enhanced relevancy along with the validity and the methodological quality of the review according to researchers. For example, PP contributions included.

‘added perspective and understanding of the study findings…broader capture and prioritization of the public’s needs’.^32^ [research team]

‘...were also able to offer commentary and critique to the themes, which supported the direction of the continuing analysis’.^29^ [research team]

‘… wanted to focus on face‐to‐face psychosocial interventions. Through discussions and good arguments, the patient representatives convinced the other group members and it was their suggestions that were followed’.^30^ [research team]

Researchers found it required diplomacy to incorporate PP contributions.

‘As a researcher it’s important to acknowledge that inconclusive and uncertain results do not indicate ineffective interventions. Rather, there is a need to be careful when interpreting the results’. (1084, PP) ‘It became very evident that we saw “non statistically different” results through very different lenses’.^30^ [research team]

Challenges to PP engagement in SRs are documented in Table 4.

**TABLE 4:** Challenges to patient partner engagement in synthesis reviews

Providing relevant, quality training to PPs.
Ensuring adequate time and resources exists to develop team relationships.
Defining and understanding team roles.
Incorporating PP suggestions constructively into the research process.
Ensuring all team members benefit from research process.
Being attentive to PP emotions.
Ensuring all team members see benefits to involvement and remain motivated to continue participation on a project.

## 4 DISCUSSION

The aim of this research was to understand how to engage PP meaningfully in SRs. Three major themes emerged from this review: the importance of fostering relationships through team development; opportunities for PP valued outcomes; and strengthen the research endeavour. The first theme provides insight both on how can research teams conduct their reviews and the characteristics of a review team that create a conducive research environment, while the latter two describe the characteristics of the research team that are conducive to meaningful contributions. Developing relationships and communication are foundational for meaningful PP engagement in SRs and the balancing of various needs. Challenges can be addressed through adequate training, clarity in communication regarding roles, allowing enough time and opportunity to build relationships, and balancing overburdening with a feeling of valued contribution. Very few of the included studies incorporate the direct voice of the PP in the processes, challenges and rewards of systematic review engagement, but among those that do, there appear to be parallels in researcher interpretation of PP participation and what the PPs experienced. However, it was the voice and reflection of PPs that address the important aspect of emotional responses, feelings of respect and desire to help others.

In the studies included in this review, PPs contributed to the SRs and received training through various mechanisms. Those mechanisms are similar to those found by Pollack et al^24^ Similarly, our themes support previous findings^22^, ^23^ in regard to the importance of relationships and research environment, clarifying expectations and roles, providing support (eg training), and PPs deriving value from involvement in research. Perhaps because of its focus on the PP perspective, our review highlighted practical and emotional elements not emphasized in either Hamilton et al or Black et al To foster partnerships in the synthesis review, PPs require accessible, respectful training that is tailored to their needs. Like any research team member, each PP brings their own unique experience and knowledge to the team but also areas in which they need to learn. Customization of training, however, is challenging for researchers leading POR review teams with diverse backgrounds. Models of meaningful engagement in research indicate that a supportive team atmosphere and research environment are essential to PP engagement in research.^22^, ^23^ This is clearly illustrated in our review by PP reflections of their experiences in SRs, which indicate that building supportive relationships between PPs and other researchers on the team is essential to ensure open communication, especially during difficult conversations. The review benefits from PP knowledge and lived experience if PP voice is heard and considered seriously. Open communication was needed to clarify expectations when PPs and researchers' expectations diverged. PPs who participated in SRs also noted a desire to be clear about their roles in the team at the beginning of the project but also throughout the duration of the project.

The results of this review expand understanding of some realities of research teams navigating various unique contributions, skill sets and knowledge, and the emotional impacts on PP with the available team resources and time. These may be specific to engagement in SRs. Our data highlighted the positive and negative emotional impacts on PPs of involvement in a synthesis review and researcher efforts to mitigate negative emotions. PP and researcher reflections indicated a need to balance being a full partner and being protected from negative emotions. Additionally, the results reveal specific ways in which the time‐consuming and lengthy synthesis review methods tax PPs and research team leaders. We noted that researchers and PPs had to navigate a line between available resources (time, work capacity) and depth of contributions, which ultimately affect the meaningfulness of engagement. Adequate resources and supports are required to permit greatest benefit from PP involvement in SRs.^36^

### 4.1 Study limitations

The COVID‐19 pandemic forced the team to pivot to a fully online mode of communication. This occurred while we were doing data extraction, but mostly impacted thematic analysis. The team acknowledged that reduced team member availability and online meeting format had the potential to significantly affect our timelines. We chose to engage CHASR to expedite the first stages of analysis. This did not adversely affect analysis quality but was a change from our planned methods.

Pollock et al^24^ commented on the limited reporting of PP involvement in systematic reviews, a concern we echo. We were often left wondering whose voice was being represented—the PP’s, the researchers’ interpretation or that of the POR team. All three voices are important to provide a complete picture of how POR review teams should be configured to permit the fullest inclusion of the PP lens in the review. However, greater clarity is needed for the reader wanting to understand which lens is being presented. Our results are a mix of these points of view, and we have tried to be clear about which voice is presented. Due to the reporting limitations, we cannot be certain we have a purely PP perspective and have had to accept this limitation to our study.

In this review, we included a variety of studies and document types as the literature exploring PP perspectives was limited. A consequence of this choice was the use of secondary data in our analysis. In‐depth qualitative data analysis was not as strong as it would be with primary data sources. Thus, we have chosen not to develop a model of PP perspectives on meaningful engagement, but rather provide some initial recommendations to guide researchers until a more robust literature is available (Table 5).

**TABLE 5:** Recommendations for how and what teams can do to support meaningful engagement of PPs in synthesis reviews

To ensure the ‘planned, supported and valued involvement of patients’,^23^ we recommend that:
PPs should be included right from inception because PP perspective can change the direction of the research. PPs opportunities to contribute be balanced with a manageable workload. PP need to know what is expected of them (and what their expectations are of the team), time commitments, and their role within the team at the outset. On‐going communication is needed to ensure clarity and permit adjustments through the project's lifespan. Training is tailored to individual PPs’ needs. Like every research team member, PP has a wide range of experiences and knowledge. Researchers need to understand this when developing relationships and preparing training. Researchers considering involving PPs in their reviews should plan for the additional time and adequate budget allocation to ensure PPs can engage in the process as fully as they desire.
To create a research environment that is conducive to effective contributions PP, we recommend:
Strong team communication. Communication is needed to create positive team dynamics in which PP can flourish, engage to their fullest extent, experience personal rewards and see the impact of their participation on health research. PP should feel comfortable presenting a different point of view and team dynamics should allow robust discussion. A significant part of this is researchers seeing PPs as possessing unique knowledge and that the POR endeavour is about joint discovery.

## 5 CONCLUSION

Meaningful PP involvement in SRs can benefit the PP and research. Fostering partnerships by taking the time for developing the team is foundational for meaningful engagement in synthesis reviews. For involvement to be meaningful for PPs, not only do team dynamics and processes need to be conducive, but PPs need to see benefits, personal or as impacts on research, from their work. There must be a sensitive balancing of various needs (eg overburdening with contributions). While this rapid review has provided preliminary guidance on how and what review teams can do, more high‐quality research about the PP perspective is needed to inform a robust model of meaningful engagement in SRs from the PP perspective.

## CONFLICT OF INTEREST

All authors declare that they have no conflict of interest.

## AUTHOR CONTRIBUTIONS

CB, AEM, TP, BB, KD, AG, DG and CS (all authors) made substantial contributions to conception and design, acquisition of data, analysis and interpretation of data of this review. All authors contributed to drafting the manuscript and gave final approval of the manuscript.

## Supporting information

Supplementary Material

## Data Availability

Data sharing is not applicable to this article as no new data were created.

## APPENDIX 1 Search strategies

### OVID MEDLINE^^^

1 Co‐researcher*.ab,ti,kf.

2 coresearcher*.ab,ti,kf.

3 ("patient‐oriented research" or "patient oriented research" or PEIR).ab,ti,kf.

4 ((patient* or consumer* or stakeholder* or citizens* or public or "knowledge user") adj3 (partner* or voice* or involve* or engag* or research* or contribut* or participat*)).ab,ti,kf.

5 Stakeholder Participation/ or Patient Participation/ or Advisory Committees/

6 1 or 2 or 3 or 4 or 5

7 ((systematic or realist or scoping or synthesis or rapid or qualitative or mixed) adj2 review*).ab,ti,kf.

8 exp "Review Literature as Topic"/ or exp Meta‐Analysis as Topic/

9 (meta‐synthesis or meta‐syntheses).ab,ti,kf.

10 ((evidence or knowledge) adj1 synthes*).ab,ti,kf.

11 integrated knowledge translation.ab,ti,kf.

12 7 or 8 or 9 or 10 or 11

13 ((perceiv* or percept* or perspective* or reflect* or experience* or voice* or view* or contribution* or empower* or 'lessons learned' or 'lessons learnt') adj3 (patient* or consumer* or stakeholder* or citizens* or public or "knowledge user")).ab,ti,kf.

14 (meaningful adj2 (engag* or participat* or contribut*)).ab,ti,kf.

15 tokenism.ab,ti,kf.

16 Perception/

17 13 or 14 or 15 or 16

18 6 and 12 and 17

19 limit 18 to yr="1998 ‐Current"

^ the two other database searches were adapted from the Medline search.

### GREY LITERATURE SEARCH

A three‐staged grey literature search was undertaken in January–February 2020, with the initial step being the identification of patient orientated research websites, likely to have relevant information on patients’ perspective of meaningful engagement in SRs. An initial list of core grey literature sources was distributed to the research team and feedback was sought based on the team's expertise and experience in this area. Additional grey literature sources were identified at this stage. As we were employing a rapid review methodology, the intent of the grey literature search was to be systematic but not exhaustive; this was similar to the approach with the database search strategy.

The second stage was a preliminary review of all the grey literature sources identified in the previous stage. This process involved a review of the website and its content to determine if a deeper investigation was warranted. A curated list of five grey literature sources was identified for further review. The sources were INVOLVE, PCORI, Healthtalk.org, NIHR Collaboration for Leadership in Applied Health Research and Care: Oxford and the CIHR Strategy for Patient‐Oriented Research and all SPOR Provincial Support Units.

The third and final stage of the grey literature search strategy was to systematically review the five identified sources to identify relevant information that met our inclusion criteria. The search strategy involved systematically browsing each organization's websites for relevant information (initial screen by title, then full‐text), as well as to complete a general site search. Any found items were cross‐referenced to our existing reference list in Covidence where it was clear that many of the grey literature sources were previously retrieved in our database search. Any unique references were added to Covidence (x = 13) and were full‐text screened.

### GOOGLE AND GOOGLE SCHOLAR

The Google search syntaxes: patient AROUND(4) "on a systematic review" and patient AROUND(4) "in a systematic review" as well as the Google Scholar Advanced Search syntax: allintitle: patient "on a systematic review" did not retrieve any novel results.
