# Supplementary material for: Patient partners’ perspectives of meaningful engagement in synthesis reviews: A patient‐oriented rapid review
Source: Health Expect. 2021 May 28;24(4):1056–71. doi: 10.1111/hex.13279 (PMC8369105; doi:10.1111/hex.13279)
Supplement: Supplementary file 1 — Supplementary Material [file HEX-24-1056-s001.docx]

**Supplementary Material I - Training on Rapid Review Methods – “Chocolate Chip Cookie” Activity**

**Format:**

This training was fully integrated into the project’s first 4-hour in-person meeting. The overarching goals of the first meeting were:

To begin to get to know one another.

To establish an open, friendly team culture.

To familiarize all team members with the basic steps of a rapid review.

To begin the process of identifying our research question.

**Activity Goal.**

The goal of the activity was to familiarize all team members with the basic steps of a rapid review employing a discipline-neutral topic familiar to all members of the team.

Our team included individuals from very different disciplinary backgrounds. We needed a common language and understanding so that the focus of the training activity was on the rapid review methods not on article content. We chose a topic everyone would be familiar with (food) and narrowed it down to chocolate chip cookies to make the example manageable.

**Pre-meeting Preparation**

**(by the Project Lead in conjunction with SCPOR personnel and one other team member).**

1. Developed the ‘Research Question’, and inclusion/exclusion criteria & forms, data extraction variables & forms – defined
2. Conducted ‘searches’ in 3 common websites with cooking recipes that had different characteristics (Epicurious.com, Sprouted Kitchen, AllRecipes.com). This yielded a ‘results set’ for screening. The cookie recipe name (‘title’), the recipe description (‘abstract’), and the full recipe (‘full text’) were downloaded.
3. Selected a subset of results from each website to ensure a sample that was small enough for the available time but also included examples that would raise questions and discussion during screening and data extraction. For instance, raising definitional issue, such as whether a skillet cookie is truly a cookie or if its size excluded it. This was designed to highlight the importance of clarity in the research question, in operational definitions, in the criteria, and in the forms. By having contentious recipes we also hoped to generate conversation about interpretation of the forms and how the team wanted to address disagreements.
4. Developed a quality assessment component. Quality assessment was a trickier topic to address, so we chose to lighten up a challenging and tiring meeting by having a taste-test for the quality assessment stage. We baked three of the cookie recipes that met criteria for ‘inclusion’ and brought them in to eat at break time. Each team member was invited to taste the cookies for ‘quality’.

**Groups.**

The team was divided into a groups of 3-4 and the Project Lead facilitated the meeting and exercises. Each group had a PP, a SCPOR stakeholder, a librarian, and an academic researcher to encourage knowledge sharing.

**Research Question.**

MATERIALS: small piece of paper to write down a name of a dessert

ACTIVITY.

1. Individuals wrote down the name of a snack
2. We shared the examples and look at the variety of dessert types and ingredients. This highlighted the many directions that could be taken even with topic as apparently focused as ‘dessert’, and lead to a discussion about the need for clarity in our research question.
3. What are the characteristics of highly ranked versus low ranked chocolate chip cookies? (this was used later)

The activity was followed by a more thorough introduction to framing research questions and a discussion of our team’s research question.

**Literature Search**

MATERIALS: search ‘results’, 30 recipes (version 1 with title & description; version 2 – with full recipe; make sure to have a mix of different kinds of recipes, some that would be challenging decisions and some weirdos; added a refid #)

REVIEW OF SEARCH PROCEDURES.

- 1. Introduction to the concepts and processes.
  2. The search had already been conducted to save time, but we reviewed the search terms (chocolate chips and cookies), the sources (epicurious, allrecipes.com, 101 cookbooks, sprouted kitchen)

**Screening**

MATERIAL: blank title/abstract and full-text forms with pre-identified inclusion/exclusion criteria.

ACTIVITU

- 1. Title & ‘Abstract’ Screening (recipes don’t have abstracts so we used the recipe description)
     1. Introduction to the concepts and processes of title/abstract screening.
     2. Individual activity – screen 5 selected titles/abstracts for inclusion/exclusion
     3. Group activity - xompare results with others within the assigned group, and as a group make a decision about how to deal with disagreements
     4. Decide which recipes will proceed to full-text screening. Give the facilitator the decisions and to receive the full-text for the next stage.
  2. Full-text Screening
     1. Introduction to the concepts and processes
     2. Individual activity – review 2 full recipes for inclusion/exclusion using the full-text screening form.
     3. Group activity - Compare results with others within the assigned group, and as a group make a decision about how to deal with disagreements
     4. Decide which recipes are included in the study. Provide the reference IDs for the included studies to the facilitator
  3. Team Discussion – how did it go? how well did you agree? were there any difficult decision and why? How did you make decisions when there was disagreement?
  4. Fill in PRISMA Flow Chart

**Data Extraction**

MATERIAL: blank data extraction form with pre-defined variables

ACTIVITY

- 1. The purpose and process of extracting data were described.
  2. Individual activity – complete the data extraction form for 1 recipe
  3. Small group activity – compare results - were there discrepancies, why? And what would you do when there are disagreements?
  4. Full group discussion - were there discrepancies, why? And what would you do when there are disagreements?

[BREAK – ‘quality’ assessment of cookie recipes by sampling the cookies]

**Analysis and Synthesis**

MATERIAL – data chart

|  | High Rank | Low Rank | Statistic |
| --- | --- | --- | --- |
| Type of Chocolate |  |  |  |
| Amount of Chocolate |  |  |  |
| Amount of Butter/other fat |  |  |  |

ACTIVITY

1. An overview of qualitative vs quantitative data, and different categories of methods for analyzing and synthesizing data were described.
2. Individual activity – fill in information in the data chart for High Rank and Low Rank
3. Small group activity – discuss the different kinds of data that might be extracted (e.g., text vs numbers), and how subset of data might be compared
4. Full group discussion – what kind of data might we be collecting and analyzing, how does this affect the types of analysis and synthesis we might do.
